# Quality of Family Relationships and Daily Stress among Middle-Aged and Older Adults

**DOI:** 10.1093/geroni/igaf122.4255

**Published:** 2025-12-31

**Authors:** Heejung Jang, David Almeida

**Affiliations:** The Pennsylvania State University, University Park, Pennsylvania, United States

## Abstract

Relationships with close family members are among an individual’s most important social bonds. Although researchers regard stress as a risk factor for eroding relationship quality, more work is needed to understand how family relationships influence exposure and affective response to daily stress. This study explores how the underlying quality of family relationships impacts exposure to daily stressors and the moderating influence of family relationship quality on the link between these stressors and negative affective responsivity. We used data from 1,236 middle-aged and older adults (Mage= 62.48 years, SD = 10.21, range 43-91; 57% female) drawn from the third waves of the Midlife in the United States and National Studies of Daily Experiences. Latent profile analysis (LPA) models identified four family relationship types: pleasant, ambivalent, neutral, and unpleasant. Multilevel mixed effects models examined the associations between these relationship types and daily stressors and tested whether family relationship types moderated the association between each type of daily stressor and negative affect. Results showed that ambivalent and neutral relationships are associated with arguments (OR = 1.86, p<.001; OR = 1.86, p<.01) and avoided arguments (OR = 1.54, p<.001; OR = 1.59, p<.01). Among participants with ambivalent relationships, avoided arguments were associated with blunted responsivity (b= -.04, p<.05). For neutral relationships, network stressors were associated with exacerbated responsivity (b= .13, p<.01). This study contributes to the literature by revealing that family relationships impact an individual’s affective responsivity to the stressors encountered in daily life.

